# 同源序列对*CYP2A13*基因SNP研究的影响

**DOI:** 10.3779/j.issn.1009-3419.2010.02.02

**Published:** 2010-02-20

**Authors:** 峰 滑, 海粟 万, 朝蓉 梅, 德杰 郑, 琳琳 孙, 军 陈, 红雨 刘, 清华 周

**Affiliations:** 300052 天津, 天津医科大学总医院, 天津市肺癌研究所, 天津市肺癌转移与肿瘤微环境重点实验室 Tianjin Key Laboratory of Lung Cancer Metastasis and Tumor Microenviroment, Tianjin Lung Cancer Institute, Tianjin Medical University General Hospital, Tianjin 300052, China

**Keywords:** 细胞色素P450代谢酶2A13, 单核苷酸多态性, 同源序列, Cytochrome P450 2A13, Single nucleotide polymorphisms, Homologous sequences

## Abstract

**背景与目的:**

已有研究表明:细胞色素P450酶2A13(cytochrome P450 2A13, *CYP2A13*)在单核苷酸多态性(single nucleotide polymorphisms, SNP)与疾病关联中起重要作用。细胞色素P450酶是一组同工酶, 基因序列间有高度的同源性, 可能对SNP分析产生影响。本研究初步探讨同源序列对*CYP2A13*基因SNP研究的影响。

**方法:**

应用Taqman探针检测573例人群rs8192789位点的分布, 采用BLAST方法分析引物结合序列。对60例样本的*CYP2A13*序列进行测序, 进一步进行了TA克隆测序, 应用BLAST方法分析了克隆测序结果。

**结果:**

rs8192789位点在573例人群中只有3例为TT纯合型, 其余均为CT杂合型, BLAST分析为同源序列导致。60例人群*CYP2A13*部分序列测序结果完全一致, 有大量套峰, 101氨基酸位点处没有dbSNP数据库中报道的SNP位点。克隆测序为247 bp、235 bp两个片段。

**结论:**

*CYP2A13*的同源序列对SNP研究造成了干扰, 部分SNP位点可能是不存在的。

## Abstract (en)

**Background and objective:**

It has been proven that cytochrome P450 enzyme 2A13(*CYP2A13*) played an important role in the association between single nucleotide polymorphisms(SNP) and human diseases.Cytochrome P450 enzymes are a group of isoenzymes, whose sequence homology may interfere with the study for SNP.The aim of this study is to explore the interference on the SNP study of *CYP2A13* caused by homologous sequences.

**Methods:**

Taqman probe was applied to detect distribution of rs8192789 sites in 573 subjects, and BLAST method was used to analyze the amplified sequences.Partial sequences of *CYP2A13* were emplified by PCR from 60 cases.The emplified sequences were TA cloned and sequenced.

**Results:**

For rs8192789 loci in 573 cases, only 3 cases were TT, while the rest were CT heterozygotes, which was caused by homologous sequences.There are a large number of overlapping peaks in identical sequences of 60 cases, and the SNP of 101 amino acid site reported in the SNP database is not found.The cloned sequences are 247 bp, 235 bp fragments.

**Conclusion:**

The homologous sequences may interfere the study for SNP of *CYP2A13*, and some SNP may not exist.

单核苷酸多态性是新一代遗传标记, 其与人类疾病的关系是遗传学研究的重要内容^[1-4]^。细胞色素P450酶2A13(cytochrome P450 2A13, *CYP2A13*)是人呼吸系统中最常见的细胞色素P450酶, 可代谢烟草中前致癌物, 因此被认为与肺癌等肿瘤的发生有关^[5, 6]^。近年有较多研究者探讨了*CYP2A13*不同单核苷酸多态性位点与肺癌的关系, 得出了有意义的结论, 使得关于*CYP2A13*单核苷酸多态性的研究成为一个活跃的领域^[7-10]^, 从相关核酸序列数据库中已经可以查到大量不同研究者报道的SNP数据。我们在研究*CYP2A13*单核苷酸多态性与肺癌关联性实验中发现了同源序列的干扰, 查阅比对了不同数据库(dbSNP、EGPsnp、HAPMAP)中的信息, 发现部分信息相互矛盾, 于是进一步做了克隆测序, 以观察同源序列是否影响SNP研究, 以期引起研究者的注意, 并能正确参考应用数据库中的数据。

## 1 材料与方法

### 1.1 研究对象

原发性肺癌266例, 正常体检人群307例, 来源于天津医科大学总医院, 均为汉族人群。收集样本人群外周静脉血2 mL, 采用Axygen外周血DNA提取试剂盒提取基因组DNA备用, 检测DNA浓度为40 μg/mL左右。

### 1.2 *CYP2A13*基因rs8192789位点(Arg257Cys)基因分型及序列比对

探针及引物由ABI公司设计合成, 上游引物:GGAGGACTTCATCGCCAAGAA, 下游引物:CGGATGAGAAAGGAGTCGATGA, VIC标记探针:AGCGTGCGCTGGTT, FAM标记探针:AGCGTGCACTGGTT。实时定量PCR反应体系为10 μL, 含探针0.25 μL, mix 5 μL, H_2_O 2.75 μL, 基因组DNA 2 μL。反应条件为:94 ℃预变性10 min, 92 ℃、15 s, 60 ℃、1 min, 共40个循环。结果判断:根据PCR曲线, 如为VIC标记探针S形曲线, 则基因型为CC型, 如为FAM标记探针S形曲线, 则基因型为TT型, 如为双重S形曲线则为CT型。检测仪器为ABI 7500 Real Time PCR system, 分析软件为SDS V1.4。每个检测单元均设立阴性对照及两个以上重复样本, 选择10%样本复测以进行实验质量控制。根据ABI公司设计引物应用BLAST进行序列比对。

### 1.3 *CYP2A13*基因序列测序分析及比对

设计上游引物:CGGACATGATGCCGTCAA, 下游引物:GAGGCCACGATGAAGGGAG AT(由赛百盛公司合成), PCR扩增反应体系:Taq(5 U/μL)0.2 μL, 10×PCR Buffer 5 μL, dNTP 5 μL, 模板DNA 2 μL, 引物各1 μL, 加灭菌蒸馏水至50 μL体系。PCR反应条件:95 ℃预变性7 min, 95 ℃变性45 s, 58 ℃退火45 s, 72 ℃延伸50 s, 72 ℃再延伸7 min, 共30个循环。采用TA克隆试剂盒p-EASY-T1 Cloning Kit(购自TransGen Biotech), 将扩增产物克隆于p-EASY-T1载体中, 转染至大肠杆菌感受态细胞, 利用蓝白斑及抗生素双重筛选, 选择30个白斑过夜摇菌后取菌液送测序。

## 2 结果

### 2.1 rs8192789位点(Arg257Cys)基因分型结果

rs8192789位点在573例人群中只有3例为TT纯合型(0.5%), 其余均为CT杂合型(图 1)。复测结果完全一致, 计算分型结果等位基因C 0.5, 等位基因T 0.5。应用BLAST比对了引物结和区序列, 发现在*CYP2A13*基因3' 64 kb处有同源序列, 同源性97%。

**1:**
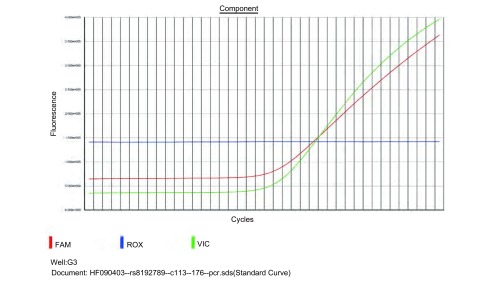
*CYP2A13*基因rs8192789位点实时荧光PCR曲线 The real time fluorescence PCR curve for the genotype of *CYP2A13* rs8192789

### 2.2 *CYP2A13*基因序列分析结果

PCR扩增目的片段247 bp(图 2), 60个样本测序完全一致, 有大量套峰, 其中101氨基酸位点处没有dbSNP数据库中报道的SNP位点(图 3)。将目的片段克隆, 随机挑取30个白斑, 摇菌后菌液送测序, 结果示247 bp、235 bp两个序列, 经BLAST分析分别为来源于*CYP2A13*、*CYP2A6*基因的序列(图 4A, B)。

**2:**
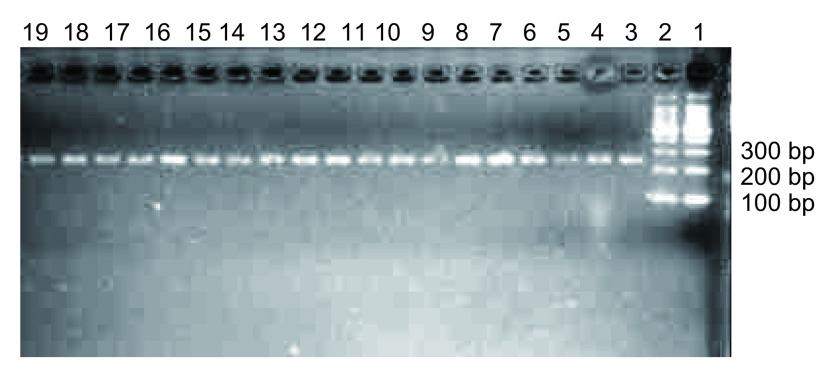
PCR扩增目的片段的琼脂糖电泳图(247 bp) Agarose gel electrophoresis of PCR products(247 bp)

**3:**
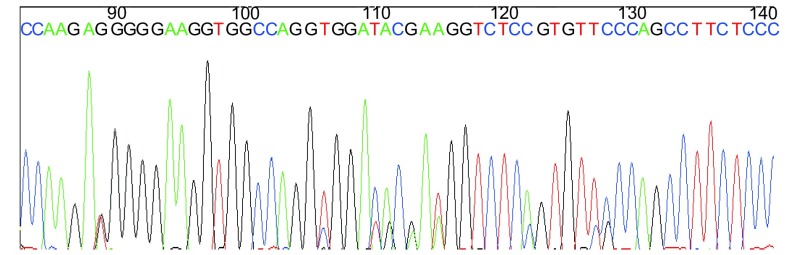
PCR产物测序图 sequence of PCR products

**4:**
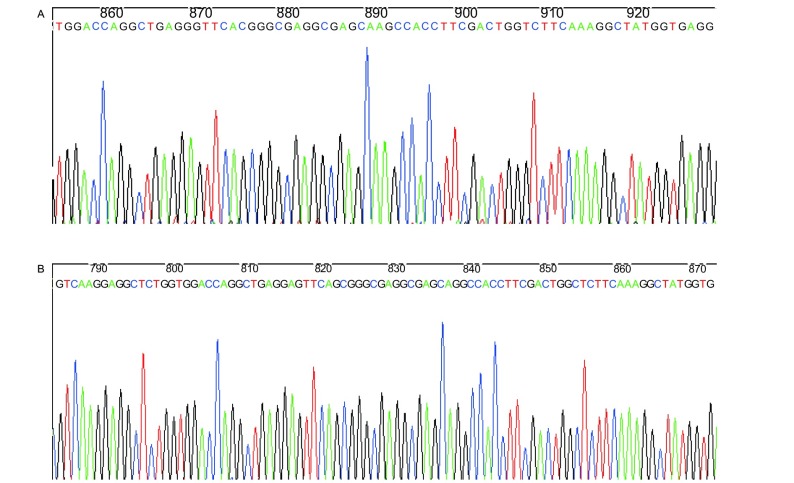
PCR产物克隆测序图 Sequence of cloned products

## 3 讨论

*CYP2A13*是P450 Ⅱ家族最新发现的一个酶, 被认为是人呼吸系统中最常见的细胞色素P450酶, 也是最主要的代谢烟草中前致癌物以及黄曲霉素的P450酶^[5, 11-14]^。关于*CYP2A13*多态性与肺癌的关联研究是当前一个活跃的领域, 从报道的结果看, 结论一致而且OR值达到2倍以上, 这表明*CYP2A13*多态性在肺癌遗传研究中非常值得关注^[7-10]^。*CYP2A13*基因位于人19号染色体长臂的CYP2A-T基因簇, 全长7.3 kb, 包括9个外显子、8个内含子, 与CYP2A6、CYP2A7序列具有高度同源性, 编码蛋白均为494个氨基酸。由于色素氧化酶P450是一大类参与内源性和外源性化合物代谢的酶, 因此*CYP2A13*与其它P450酶也起码具有40%以上的序列同源性^[15]^。关于*CYP2A13*单核苷酸公布的数据, 在dbSNP数据库中上传了139个SNP位点, Hapmap数据库于2009年2月公布的最新数据报告了6个SNP位点, 全部包括在dbSNP中, EGPSNP数据库暂未收录该基因数据。

我们选择rs8192789位点定制了Taqman探针, 该位点位于257位氨基酸位点, 密码子的第一个碱基为T/C, 对应氨基酸分别为色氨酸、精氨酸。该位点在dbSNP数据库中由Hapmap上传的数据为单一的CT杂合子, 而该位点在Hapmap数据库2009年2月公布的最新数据中已经删除。Wang^[9]^利用限制性酶切的方法研究了791例中国汉族正常人群rs8192789位点的基因型频率, 其中CC、CT、TT频率分别为82.4%、16.4%、1.2%。我们分型的结果是单一的CT杂合子, 同dbSNP数据库中的频率是一致的, 从遗传的角度来讲, 这种基因型分布实际上是不可能的。利用引物结合区域序列进行了BLAST比对, 发觉引物结合序列同位于该基因簇的一段假基因有97%同源性, 我们认为是该同源序列导致的。虽然Hapmap更新的数据库删除了rs8192789位点, 但我们认为这一位点SNP是应该存在的。60例样本人群的*CYP2A13*序列测序完全一致, 有大量套峰集中分布, 包括了dbSNP数据中的rs3826712位点, 进一步将序列进行了克隆测序, 结果表明为CYP2A6干扰所致, 我们认为rs3826712位点可能是不存在的, 另外dbSNP数据库中也没有发现rs72552266位点。

造成SNP假阳性的原因除了分型技术误差外, 另一个原因就是由于同源序列导致的干扰, 这也可能是导致推断人类SNP出现频率差异的主要原因。通过对*CYP2A13*序列的初步分析, 我们认为在dbSNP数据库中的139个SNP位点部分是由于同源序列导致的假阳性的结果。Hapmap数据库中包含了中国汉族人群的SNP分布, 是研究SNP重点参考的数据库^[16]^, 但由于工作量庞大, 其发布的数据可能也会有疏漏, 因此我们在参考这些相关数据时要重点考虑到同源序列导致的对结果的干扰, 以便正确参考应用相关数据。
